# Analysis and actions after laboratory errors in a Chinese university hospital

**DOI:** 10.1186/s12913-025-13320-5

**Published:** 2025-10-03

**Authors:** Ying Guo, Wei Dai, Yongmei Jiang, Xiaojuan Liu

**Affiliations:** 1 Department of Laboratory Medicine, West China Second University Hospital, Sichuan University, No. 20, Section 3, Ren Min Nan Lu, Chengdu, 610041 Sichuan P.R. China; 2 Key laboratory of Birth Defects and Related Diseases of Women and Children (Sichuan University), Ministry of Education, Chengdu, P.R. China

**Keywords:** Laboratory-related errors, Quality improvement, Corrective action

## Abstract

**Background:**

Diagnostic errors pose a critical threat to patient safety, heavily relying on accurate laboratory medicine. However, research specifically addressing laboratory errors (LEs) remains limited globally. This study aimed to categorize LEs, identify their root causes, and develop targeted interventions within a large, specialized hospital in China, where systemic factors amplify their potential impact.

**Methods:**

A retrospective quality improvement study was conducted in the ISO 15,189 and CAP-accredited Department of Medical Laboratory at a women and children’s hospital. Eighty-three documented LEs (51 general, 32 transfusion-specific) from March 2016 to April 2023 were analyzed. Errors were captured via internal incident reporting and hospital risk management systems. LEs were classified using five criteria: responsibility attribution (exclusively lab, extra-lab, conjoint, undetermined), testing phase (preanalytical, analytical, postanalytical), error type, preventability (using a cognitive psychology framework: cognitive vs. noncognitive), and patient impact. Root cause analysis and corrective actions were tracked.

**Results:**

Among the 51 general LEs, the preanalytical phase was most error-prone (51.0%), primarily due to specimen collection (29%) and request procedure errors (22%). Analytical (4%) and postanalytical (18%) phases had fewer errors. Responsibility analysis showed 20% exclusively lab-originated, 60% extra-lab-originated, and 16% conjoint. Cognitive errors dominated preventable incidents. Environmental/infrastructure (6%) and Laboratory Information System (LIS) errors (14%) were significant concerns. Separately, among 32 transfusion-related errors, clinical physicians bore primary responsibility in 51%, with common issues being improper specimen collection (22%) and non-evidence-based orders (16%). Corrective actions (e.g., workflow optimization, staff training, improved communication, LIS upgrades like an electronic critical value notification system, facility relocation) led to significant reductions in preanalytical errors over time. Improvements were achieved cost-effectively.

**Conclusion:**

Preanalytical errors are the most prevalent LEs, often originating outside the laboratory. Cognitive errors are highly preventable. Implementing targeted interventions based on systematic error classification and root cause analysis—including technological solutions (e.g., electronic alerts, LIS improvements), workflow simplification, enhanced training (especially for non-laboratory personnel in transfusion contexts), and interdepartmental communication—significantly reduces LEs and enhances laboratory quality management. Continuous monitoring and context-specific strategies are crucial, especially in large healthcare systems. Study limitations include potential underreporting and limited generalizability beyond specialized women and children’s hospital settings.

**Supplementary Information:**

The online version contains supplementary material available at 10.1186/s12913-025-13320-5.

## What is already known on this topic

The preanalytical phase as the predominant source of LEs, often originating from clinical workflows (e.g., test ordering, specimen collection). Transfusion-related LEs pose unique risks due to multispecialty involvement and potential for severe outcomes. Despite this recognition, systematic analyses of error attribution and context-specific interventions remain limited.

## What this study adds

This study establishes a dual classification framework integrating cognitive psychology principles (distinguishing cognitive vs. non-cognitive errors) with the entire inspection process enabling precision-targeted interventions for laboratory errors (LEs) mitigation. Implementation of a closed-loop electronic critical value notification system and workflow optimizations significantly reduced errors.

## How this study might affect research, practice, or policy

This work validates a novel dual taxonomy (cognitive vs. non-cognitive × testing phase) for laboratory error analysis, establishing a standardized framework for future investigations into systemic diagnostic failures and transfusion-specific safety gaps. It catalyzes clinical-laboratory integration through non-laboratory personnel training, PDA-based specimen verification, and closed-loop critical value reporting systems—demonstrating cost-effective solutions adaptable to resource-constrained settings.

## Introduction

Diagnostic errors, resulting from a complex interplay of systemic and cognitive factors with often multiple root causes, represent a critical threat to patient safety and healthcare quality [1]. Laboratory medicine plays a fundamental role in mitigating this risk, as diagnostic accuracy heavily relies on its outputs. This discipline, which focuses on the quantitative measurement and qualitative assessment of analytes in biological fluids [2], provides essential data for clinical decision-making. Consequently, errors within the laboratory process—defined as “a defect occurring at any stage of the laboratory cycle, from test ordering to result reporting and the appropriate interpretation and response to these results” [3]—pose a significant risk to diagnostic integrity. Given the critical dependence of diagnosis on laboratory information, rigorous scrutiny of such errors is imperative. This scrutiny is particularly vital in large, rapidly evolving healthcare systems like China’s, where a massive population and ongoing health transitions amplify the potential impact of laboratory errors (LEs) on patient safety and healthcare quality.

Although there have been studies on laboratory errors worldwide [4–6], similar research is relatively scarce in China. To address this gap, this study draws upon the reasons and solutions for LEs identified within the laboratory department of West China Second University Hospital of Sichuan University in China over the past eight years. The insights gained aim to serve as a guide for enhancing laboratory quality management. Review of these documented events prompts the discussion of effective interventions to reduce or eliminate such errors.

## Materials and methods

### Study site and aim

We conducted a quality improvement study in the Department of Medical Laboratory at a specialized women and children’s hospital that encompasses 31 clinical departments. As the first laboratory among China’s specialized hospitals to be accredited by both ISO 15189 (2006) and the College of American Pathologists (CAP) (2013), our department offers a comprehensive range of testing services, including clinical chemistry, hematology, immunology, microbiology, molecular diagnostics, and transfusion medicine. The primary objectives of this study were to: a). Categorize LEs; b). Identify types of LEs and their root causes; c). Develop measures to reduce LEs.

### Data charting and analysis

#### Error reports database

This retrospective study analyzed 83 laboratory-related errors documented from March 2016 to April 2023. Errors were captured through the laboratory’s internal incident reporting system and the hospital’s risk management system. All laboratory staff are mandated to report incidents that significantly impact patients and are encouraged to report all errors using standardized forms that include: (1) Event time, location, and department; (2) Process description and investigation findings; (3) Preventive assessments and corrective actions; (4) Evaluation of patient impact. The included errors encompassed all aspects of laboratory services: (1) Testing procedures (including transfusions); (2) Physical facility issues; (3) Patient service complaints.

#### Classification of laboratory errors

LEs were classified based on five criteria: (1) responsibility attribution, (2) testing phase (preanalytical, analytical, or postanalytical), (3) error type, (4) preventability, and (5) patient impact. Transfusion-specific errors, including blood group, antibody screening, and crossmatching, were analyzed separately due to their specialized nature. For the assessment of preventability, medical risk managers and laboratory quality managers conducted a joint evaluation of preventability and patient impact. To address frequent disagreements—often stemming from physicians attributing errors to systemic laboratory failures—we implemented a cognitive psychology framework [7]. This framework distinguishes between noncognitive errors, which are unintentional deviations from automated procedures (e.g., mislabeling specimens due to lapses in attention), and cognitive errors, which arise from knowledge gaps or flawed information processing (e.g., inappropriate test selection due to misinterpreted guidelines). This dual classification facilitates targeted interventions while standardizing preventability judgments.

#### Responsible party of laboratory errors

The responsibilities were classified into mutually exclusive categories: (a) exclusively laboratory-originated, indicating that the root cause lies within laboratory-controlled processes; and (b) extra laboratory-originated, which involves personnel, equipment, or services beyond the governance of the laboratory. (c) Conjoint responsibility: laboratory protocols were executed correctly, indicating a conjoint responsibility. The causal chains involved interdependent laboratory and clinical factors, with a responsibility share exceeding 30% per domain. (d) Unable to determine: in cases where evidence was insufficient, the responsibility distribution was deemed indeterminate. Final determinations of responsibility required operational validation through an independent review by the laboratory director and quality manager. This structured approach facilitated the systematic mapping of error origins and the distribution of responsibilities across healthcare subsystems.

## Results

### Laboratory error incidents

#### Overview and examples of laboratory errors

A total of fifty-one LEs occurred at the department of laboratory medicine from 2016 to 2023. Incident types are depicted in Table 1. Of the LEs in phase of testing, 26 (51.0%) involved in the preanalytical phase, 2 (4%) the analytic phase, and 9 (18%) the postanalytical phase. The most common preanalytical problems involved specimen collection (29%) and request procedure error (22%). Two LEs occurred during the analysis phase, primarily involving instrument/equipment failures and water circulator malfunction during inspection. These delays affected timely report issuance. Nine laboratory errors (18% of the total errors) occurred during the post-analytical phase, including failures in clinician-result communication, issuance of incorrect reports, and unverified critical values. Laboratory errors related to environmental conditions, facility infrastructure, and information systems are significant concerns. Regarding environmental facilities, three cases (6% of incidents) of occupational contact dermatitis were reported among medical staff due to non-compliant medical supplies, such as gloves. Additionally, one incident of syncope, secondary to heat exhaustion, was documented in a patient awaiting blood collection, which was attributable to the absence of cooling equipment in the waiting area. Seven errors (14% of incidents) were specifically linked to the laboratory information system (LIS). For classification purposes, we follow this principle: Errors arising from human factors, such as the failure to receive specimens or modify specimen types, are categorized as pre-analytical errors. In contrast, failures associated with the LIS, including unsuccessful specimen accessioning and misidentification of specimen types or patient information, are classified as information system errors.

**Table 1:** Overview of Laboratory-related errors

Error type	Sub-error type	Defects found (No., %)
Preanalytical		
Request procedure error	Sample without order, duplicate order, missing/incorrect patient ID, incorrect time/date	11(22)
Specimen collection	Inappropriate container, incorrect temperature	15(29)
Analytical		
Regent-caused error	Change batch of regent, expired reagent, other	1(2)
Analyze interference	Hemolysis, Icterus, Heterophile antibodies, drugs, other	0(0)
Apparatus	Water circulator malfunction, power failure, other	1(2)
Postanalytical		
Process	Lack of communication within laboratory	3(6)
Reporting	Incorrected result, incorrected report template information	4(8)
Delivering	Critical value not confirmed, TAT excessive	2(4)
Physical Facilities		
Events related to the laboratory environment	Patients experienced heatstroke due to elevated temperature in the blood collection facility	1(2)
Events related to the laboratory regents	Reagent inventory and usage were not estimated correctly	0(0)
Events related to the laboratory facilities and supplies	Allergic skin due to inferior medical supplies	3(6)
Laboratory Information and Communication		
Check-in of specimens	Specimens were lost due to lack of proper checked-in	1(2)
Billing issues	The patient’s treatment was not billed due to issues with the information system	3(6)
Transmission issues	LIS transmitted incorrect or untransmuted results	1 (2)
Routine operation failure	The sample type is immutable and cannot be altered	2 (4)
Unable to determine	/	3(6)

#### Responsibility for the incidents

For incidents responsibility, out of fifty-one incidents, 20% were solely attributed to the laboratory while 60%involved a component beyond the laboratory’s jurisdiction (Table 2). 16% incidents were attributed to the laboratory and other department. Table 4S presents the specific laboratory subgroups involved in ten laboratory errors for which the laboratory was responsible. As anticipated, pre-analytical sample processing errors constituted the largest proportion, accounting for 30% of laboratory-attributable incidents. Table 3 illustrates the process of these error identifications. Self-reflection and self-improvement of our laboratory were the main sources of errors, accounting for 77%.

**Table 2:** Potential impact of fifty-one laboratory errors on patients and their classification by responsible party with examples

Category	Potential impact on patients	Examples	No. (%)
Responsible Party			
Laboratory only	/	Reagent inventory and usage were not estimated correctly	10(20)
Outside laboratory	/	The limited availability of outpatient services and the absence of a convenient clinic make it difficult for non-local patients to complete their diagnosis and treatment.	30(60)
Laboratory and Outside laboratory	/	The gynecological clinic was not staffed on weekends, resulting in no personnel available to respond to critical values.	8(16)
Unable to determine	/	The patient altered the test report for personal gain	3(6)

/: No actual harm occurred to the patient(s)

**Table 3:** Responsible party and sources of fifty-one laboratory-associated errors

Responsible Party/Sources (*n*, %)	Self-reflection and self-improvement	Clinicians’ complaints’	Patient grievances	Others
Laboratory only	4(8)	3(6)	2(4)	1(2)
Outside laboratory	28(55)	0(0)	0(0)	2(4)
Laboratory and Outside laboratory	5(10)	2(4)	1(2)	0(0)
Unable to determine	2(4)	0(0)	1(2)	0(0)

### Preventability of the incidents

The classification of the fifty-one incidents by preventability is presented in Table 4. Among the preventable events, cognitive errors were more prevalent than non-cognitive errors, with three error events involving both cognitive and non-cognitive causes. One unpreventable event was attributed to a failure of the Laboratory Information System (LIS), an incident that is typically unforeseeable.

**Table 4:** Classification of fifty-one error reports related to laboratory by their preventability

Preventability	Error Category	Examples	No. (%)
Yes	Noncognitive error	Failure to check the patient’s information before blood drawing; Check-in not performed (in the Laboratory Information Systems; Failure to implement a proper handover protocol following specimen collection resulted in the loss of specimens; Erroneous test reports were issued due to the failure of laboratory personnel to verify test results.	17(33)
	Cognitive error	The lack of a specialized swab for routine leucorrhea sampling hinders detection speed and leads to instrument failure; wastage of plasma occurred owing to lack of knowledge about whether RhD negative plasma can be safely transfused to Rh positive patients; The incongruity between point-of-care (POCT) and venous blood glucose readings has led physicians to base treatment on POCT values.	10(20)
	Cognitive and noncognitive error	Critical value notifications went unanswered(noncognitive) at the gynecological clinic on weekends due to a lack of staffing (Cognitive);	3(6)
No	/	Software error of LIS receiving server in Information department caused “receiving time” in the laboratory report to be earlier than “sampling time”	1(2)
Unable to determine	/	The patient illicitly utilized another individual’s medical card; Patients falsified the test results for personal gain	20(39)

### Transfusion error incidents

#### Laboratory errors related to transfusion

Laboratory errors related to transfusion must not be overlooked. The overview of incident description of the thirty-two transfusions related LEs is given in Table 5. Among 32 transfusion-related laboratory error incidents, primary responsibility was attributed to clinical physicians in 51% of cases. Laboratory errors were implicated in 25% of incidents, with the remaining 25% being uncategorizable. Clinician involvement was documented in 16 cases (50%), whereas laboratory personnel involvement was identified in 8 cases (25%). In 22% of errors, improper crossmatch specimen collection was precipitated by unauthenticated patient identification. Additionally, 16% of incidents were triggered by non-evidence-based transfusion orders, resulting in preventable product wastage. The laboratory quality management team also assessed the consequences of LEs related to transfusion, as described in Table 5.

**Table 5:** Hotspots for errors in thirty-two transfusion laboratory testing

Responsible Party	Event Description	Consequences of Laboratory errors (e.g. Temporary or permanent harm, death, etc.)	No. (%)
The clinician	Cross-matching sample collection errors occurred without verifying patient information or medical orders.	Puncture blood vessels repeatedly	7(22)
	Incorrect requests for cross-matching	Increased medical expenditures for patients	3(10)
	Blood products are often unnecessarily requested by clinicians, resulting in the wastage of valuable resources.	The overutilization of medical resources exacerbates the financial burden on patients.	5(16)
	Administration of fresh frozen plasma to preterm infants with low birth weight resulted in the production of non-endogenous and anomalous antibodies	Regular treatment regimens were made more difficult by the patient’s abnormal antibodies, but no negative effects were observed.	1(3)
The laboratory	The newborn’s blood type was incorrectly recorded as type B due to a clerical error, resulting in an inaccurate blood typing for the patient.	Erroneous inspection report was found out in time before treatment and do not cause any harm for patient.	2(6)
	Physicians who checked the blood products found that the patient’s information did not match that on the blood bag.	Errors were corrected in time before occurring and do not cause any harm to patients.	2(6)
	Errors in the collection of cross-matched blood samples by clinicians led to the issuance of erroneous reports	Errors were corrected in time before occurring and do not cause any harm to patients.	4(13)
Others	The medical order status of blood transfusion may be modified at the discretion of healthcare professionals following discharge from hospital. LIS issues caused a delay in receiving cross-matching results. Unable to determine	An increased economic burden on patients and do not cause any harm to patients. No significant effects on patient care /	4(13) 2(6) 2(6)

### Key elements of responses and actions after laboratory errors

Table 6 summarizes the corrective actions implemented following laboratory error incidents over the 8-year study period. The theme of actions for LEs was comprised of four elements: recognize laboratory errors, communicate and clarify the issues exposed, targeted improvement measures and effect observation.

**Table 6:** Key elements of responses and actions after laboratory errors regarding the time of occurrence

Year	No. (%)	Exposure concerns	Preventative measures
2016	21(25)	1. Patient information and order information were not checked before sampling. 2. The staff exhibited insufficient care and attentiveness and insufficiency in professional expertise	a. Standardization of the blood drawing process. b. To optimize workflows and engage the hospital’s medical department in coordination c. To provide opportunities for continuous education.
2017	13(16)	1. Insufficiency of clinicians’ professional knowledge about blood transfusions. 2. Technical problems of laboratory information system. 3. Poor service attitude caused patient dissatisfaction	a. Update and upgrade laboratory information system, arrange resident information personnel to solve information communication problems. b. Improved the service attitude of our staff by training, supervision, assessment, and complaint management
2018	6(7)	1. Insufficiency of clinicians’ professional knowledge about blood transfusions. 2. Patient information and order were not checked	a. In-depth communication with clinicians on the management and application of blood products
2019	6(7)	1. Inadequate level of digitalization, and the insufficient development of LIS functionality. 2. Inadequate and inconveniently laboratory space.	a. Electronic management of reagent consumption and inventory, with real-time monitoring. b. Relocate the laboratory waiting room
2020	22(27)	1. LIS lacks timely updates, resulting in a high failure rate. 2. Unfamiliar with medical order items by the doctors. 3. Inadequate procurement and handling of medical supplies and equipment	a. Update and upgrade laboratory information system, arrange resident information personnel to solve information communication problems. b. To increase the publicity of testing items and medical orders for clinicians. c. Acquisition and acceptance of medical supplies is closely supervised
2021	7(8)	1. Insufficiency of clinicians’ professional knowledge about blood transfusions. 2. Inadequately communication with other departments about transfer of specimens	a. In-depth communication with clinicians on the management and application of blood products. b. To adjust workflows; and invite the hospital’s medical department to coordinate communication;
2022	5(6.0)	1. The staff exhibited insufficient care and attentiveness. 2. The procedure of venipuncture lacked standardization	a. Standardization of the blood drawing process and strengthened training. b. To clear job responsibilities and adjust workflows
2023	3(4)	1. Blood gas analysis kit was not replaced promptly 2. Values of POCT were inconsistent with the biochemical analyzer	a. Acquire a supplementary blood gas analyzer as contingency equipment b. Comparative trials were conducted, and any discrepancies in results were discussed with the physician

Most LEs occurred in 2016(25%) and 2020 (27%). Problems such as clinicians not being familiar with the medical orders of laboratory test items and punctures without checking patient information by blood collection personnel surfaced. After the root-cause analysis by the team, we overall have taken measures such as optimizing work processes, increasing staff training and assessment, improving staff service attitudes, strengthening communication with clinics and publicizing projects. The decrease in preanalytical issues was more significant than the decrease in other error types. Over time, potential problems with laboratory environmental facilities and information systems became apparent. Improvements such as the updating of information systems, the move to more spacious laboratory, and the implementation of electronic information management have to some extent addressed these potential problems. These effects were achieved through an extremely low-cost feedback initiative, highlighting that our improvement measures would be suitable for implementing across healthcare environments with limited resources.

## Discussion

In this quality improvement study, we retrospectively analyzed LEs in our laboratory across all aspects of laboratory quality management which are essential for the implementation and evaluation of the improvement strategies based on laboratory accreditation criteria to reduce their occurrence and patient harm. This study showed that continuous quality improvement based on the laboratory-related errors can help a laboratory discover the invisible part of the iceberg hidden in the darkness of errors. As we strived for even higher quality, it became apparent that the majority of errors in the testing process are found in the preanalytical phase which exerts a strong influence on laboratory testing and clinical outcomes and may severely jeopardize patient safety, which is a major contributor to diagnostic error. The specific results obtained in our study are similar to those of studies which based on laboratories regarding the phase of laboratory testing most frequently involved in errors [8–14].Complex workflows of preanalytical phase are prone to mistakes. The main causes of these errors were directly related to the sample collection process. Once an error occurs, the first step is to identify the existence of these errors as well as their types and frequencies [15, 16]. Then, appropriate risk management strategies should be implemented to improve quality and avoid LEs that jeopardize patient safety. Corrective actions such as strengthening training, assessment and supervision probably can reduce the likelihood of employees making mistakes, thereby reducing the occurrence of errors. Examples include addressing issues such as an experienced phlebotomist forgetting to enter a sample in the system during the preanalytical phase or failing to deliver samples on time for testing. In such cases, simply providing additional training, assessment, or supervision are often not the most effective interventions. Instead, a more appropriate approach focuses on identifying and addressing the root causes of these errors. We tailor improvement strategies based on the nature of the error: For errors caused by cognitive errors (e.g., misunderstanding a new procedure), interventions like targeted training and enhanced supervision may be effective. For errors arising from non-cognitive errors, (e.g., overlooking a step due to complexity or time pressure), we prioritize optimizing workflows, reducing work complexity and volume, and implementing technological safeguards. For instance: Strengthening communication with clinics and clarifying testing protocols can reduce LEs related to duplicate or incorrect orders, often rooted in systemic communication issues or unclear procedures. Implementing a barcode/PDA entry system provides a technological solution to significantly reduce errors of omitted specimen check-in, addressing a failure point prone to human oversight within a complex process. Hence, a range of targeted measures have been implemented to enhance quality and ensure continuous improvement in our lab [17], including increasing the number of specimen transporters, purchasing new instruments, and optimizing information systems. The outcomes of these improvements have been satisfactory. Automation and standardization remain crucial strategies for minimizing pre-analytical errors. Implementing specific corrective actions, such as optimizing transportation processes and upgrading information systems, is an effective approach. By standardizing, monitoring, and evaluating quality indicators (QIs) throughout the laboratory’s pre-analytical stage, laboratories can enhance quality, reduce errors, and ensure patient safety through data-driven methodologies [18]. Consequently, in our subsequent work, we will adopt clearly defined QIs and establish an appropriate data collection strategy to identify, monitor, and effectively assess critical laboratory activities and errors.

A critical contributing factor to laboratory errors (LEs) during the post-analytical phase was the reporting of critical values to clinicians without timely confirmation. To address this issue, we developed and implemented an electronic critical value notification system. This system automatically prompts physicians to confirm receipt after notification and triggers a callback from laboratory staff if confirmation is delayed. This closed-loop process is designed to fundamentally resolve issues associated with unconfirmed critical value reporting.

As the transfusion laboratory is administered by the Department of Laboratory Medicine, we have broadened our focus to include transfusion-related laboratory errors. Transfusion is a complex, multistep process that involves various professional groups, including physicians, nurses, laboratory scientists, donors, and recipients. Errors can occur at any stage, potentially jeopardizing patient safety. These mistakes often arise from the omission of critical checks, such as taking shortcuts, or from the assumption that safety responsibilities are delegated to others. Clinicians perceive patient blood management as the process of transfusing the appropriate blood product to the correct patient at the optimal time [19]. In contrast, according to our error data, blood bank staff primarily identify sample collection errors and the wastage of blood products as the main concerns. This issue is particularly prevalent in cross-matching and blood group antibody screening, where specimens are primarily collected by non-laboratory staff, such as nurses, who lack training in verifying patient identity prior to specimen collection, as stipulated in CAP-accredited guidelines [20]. This error can seriously influence the patient life safety and can also result in serious outcomes in blood transfusion. Otherwise, this error also reveals a lack of general knowledge and awareness of blood drawing which is completely caused by human factors and should thus be reduced. Education and training on the blood drawing procedures which including preparation for patients, use of appropriate containers, correct puncture, check labels, and criteria for sample rejection was urgently needed for nonlaboratory personnel. Our professional phlebotomists group helps them to reduce patients’ suffering and blood hemolysis due to repetitive vein punctures. Meanwhile, we strongly demand every phlebotomist to sign the sample tubes after drawing blood, which enables us to trace the source of the disqualification samples and determine why the error occurred.

This study has several limitations inherent to its retrospective, descriptive design and specific institutional context. The analysis of documented laboratory error events over an 8-year period, by its nature, makes it difficult to establish causal relationships or draw definitive conclusions. The relatively limited dataset—documenting only 83 error events over this eight-year period—likely underestimates the true incidence rate. This underestimation may stem from underreporting due to staff hesitancy (e.g., concerns about accountability) and systemic constraints, such as the absence of dedicated electronic systems for error reporting and evaluation. Furthermore, the original data comprise qualitative descriptive reports, making it challenging to apply statistical measures for quantification. Additionally, although transfusion testing is often managed separately from core laboratory functions due to its specialized nature, it falls under the unified management of our Department of Laboratory Medicine. Therefore, transfusion-related errors were included in this analysis. Future studies, however, could benefit from examining these errors separately where feasible. Finally, the specialized setting of a Women and Children’s Hospital, with a patient population predominantly comprising pregnant/postpartum women and children and corresponding sample types, may limit the generalizability of the findings to broader hospital settings or different patient demographics.

In conclusion, the accurate identification of any laboratory errors that have occurred or may occur plays a crucial role in maximizing quality in clinical laboratories. The findings of this study should serve as a bellwether for identifying error-prone domains in laboratory medicine, which can facilitate the development of appropriate quality improvement initiatives.

## Supplementary Information

Supplementary Material 1.

## Data Availability

No datasets were generated or analysed during the current study.

## Abbreviations

LEs: Laboratory errors
CAP: College of American Pathologists
