# Supplementary material for: Analysis and actions after laboratory errors in a Chinese university hospital
Source: BMC Health Serv Res. 2025 Oct 3;25:1296. doi: 10.1186/s12913-025-13320-5 (PMC12495776; doi:10.1186/s12913-025-13320-5)
Supplement: Supplementary file 1 — Supplementary Material 1. [file 12913_2025_13320_MOESM1_ESM.docx]

**Table 2 S Specific laboratory subgroup involved in ten Laboratory errors in which the laboratory was responsible for the events**

| **Specific laboratory subgroup** | **Other departments** | **Example** | **No. (%)** |
| --- | --- | --- | --- |
| Preanalytical group | / | The elevated temperature within the blood collection room resulted in patients awaiting phlebotomy experiencing heatstroke | 3(30) |
| Microbiology | Pediatric Cardiovascular Department | The technologist erroneously recorded the results of antimicrobial susceptibility testing in the patient's medical record. | 2(20) |
| Phlebotomy | Outpatients/Gynecological inpatients | Specimen collection errors occurred due to the phlebotomist 's failure to verify the patient's information. | 1(10) |
| Hematology | Pediatric Hematology Department | The laboratory's notification of critical values was not consistently synchronized with clinical recording. | 2(20) |
| Chemistry | Information Department | The implementation of the remote-control system resulted in communication disruptions and significant delays in specimen testing and data transmission for the biochemistry.  Test results were copied incorrectly, and an incorrect report was issued | 2(20) |
